# Garcinol sensitizes human head and neck carcinoma to cisplatin in a xenograft mouse model despite downregulation of proliferative biomarkers

**DOI:** 10.18632/oncotarget.2881

**Published:** 2015-02-28

**Authors:** Feng Li, Muthu K. Shanmugam, Kodappully Sivaraman Siveen, Fan Wang, Tina H. Ong, Ser Yue Loo, Mahadeva M.M. Swamy, Somnath Mandal, Alan Prem Kumar, Boon Cher Goh, Tapas Kundu, Kwang Seok Ahn, Ling Zhi Wang, Kam Man Hui, Gautam Sethi

**Affiliations:** ^1^ Department of Pharmacology, Yong Loo Lin School of Medicine, National University of Singapore, Singapore; ^2^ Cancer Science Institute of Singapore, Centre for Translational Medicine, Singapore; ^3^ Division of Cellular and Molecular Research, Humphrey Oei Institute of Cancer Research, National Cancer Centre, Singapore; ^4^ Genome Institute of Singapore, Agency for Science, Technology and Research (A*STAR), Singapore; ^5^ Jawaharlal Nehru Centre for Advanced Scientific Research, Molecular Biology and Genetics Unit, Transcription and Disease Laboratory, Bangalore, India; ^6^ School of Biomedical Sciences, Faculty of Health Sciences, Curtin University, Western Australia, Australia; ^7^ Department of Biological Sciences, University of North Texas, Denton, Texas, USA; ^8^ Department of Haematology-Oncology, National University Health System, Singapore; ^9^ College of Korean Medicine, Kyung Hee University, Seoul, Republic of Korea

**Keywords:** HNSCC, chemoresistance, NF-κB, proliferation, garcinol

## Abstract

Platinum compounds such as cisplatin and carboplatin are frequently used as the first-line chemotherapy for the treatment of the head and neck squamous cell carcinoma (HNSCC). In the present study, we investigated whether garcinol, a polyisoprenylated benzophenone can chemosensitize HNSCC to cisplatin. We found that garcinol inhibited the viability of a panel of diverse HNSCC cell lines, enhanced the apoptotic effect of cisplatin, suppressed constitutive as well as cisplatin-induced NF-κB activation, and downregulated the expression of various oncogenic gene products (cyclin D1, Bcl-2, survivin and VEGF). *In vivo* study showed that administration of garcinol alone (0.5 mg/kg body weight, i.p. five times/week) significantly suppressed the growth of the tumor, and this effect was further increased by cisplatin. Both the markers of proliferation index (Ki-67) and microvessel density (CD31) were downregulated in tumor tissues by the combination of cisplatin and garcinol. The pharmacokinetic results of garcinol indicated that good systemic exposure was achievable after i.p. administration of garcinol at 0.5 mg/kg and 2 mg/kg with mean peak concentration (Cmax) of 1825.4 and 6635.7 nM in the mouse serum, respectively. Overall, our results suggest that garcinol can indeed potentiate the effects of cisplatin by negative regulation of various inflammatory and proliferative biomarkers.

## INTRODUCTION

Despite advances in earlier detection and novel therapies for HNSCC, it still remains a major health concern and causes more than 350,000 deaths annually worldwide [1]. Multidisciplinary approaches involving surgical excision, chemotherapy, and radiation therapy are frequently used for the treatment of HNSCC, but have shown limited efficacy due to tumor recurrence, drug resistance, and distant metastases in locoregionally advanced disease [2]. The chemotherapeutic drug, cisplatin (*cis*-dichloro-diammineplatinum (II)) has been approved by FDA for the treatment of HNSCC since 1970s, and still remains as the standard and first-line chemotherapy for HNSCC treatment although many other agents have also been subsequently developed [3, 4]. A meta-analysis of a panel of clinical studies has revealed that platinum containing regimens commonly exhibit higher response rates than non-platinum therapies [5]. Unfortunately, only 20 to 40% of the patients experience complete response to platinating agents, mainly because of the development of intrinsic or acquired resistance to chemotherapy [6]. High doses of platinating agents which are required for significant anti-tumor effect frequently lead to severe side effects such as nausea, vomiting, mucositis, neurotoxicity, and renal dysfunction that compromise the quality of life of the patients [7, 8]. For the above reasons, novel pharmacological agents with lower toxicity which can enhance the effects of existing drugs are in great need.

Several studies have previously documented the prevalence of constitutive activated NF-κB in diverse HNSCC cell lines and tumor tissues specimens [9–12], and this dysregulated NF-κB signaling contributes to the cell survival, angiogenesis, migration, tumorigenesis, and therapeutic resistance of HNSCC [13–15]. Global gene profiling analysis has also clearly indicated that NF-κB signaling is a major contributor to metastatic progression of HNSCC [16] and a prognostic biomarker of a high-risk disease [17]. Elevated phosphorylation level of NF-κB in patient samples is often associated with poor prognosis in terms of high recurrence and poor survival [18]. Additional studies have shown that several chemotherapeutic agents including platinum compounds can induce NF-κB activation signaling through diverse molecular mechanism(s) in various tumor cell types including HNSCC [19–24]. For example, cisplatin has been shown to significantly induce NF-κB promoter activity, accompanied with the increase in nuclear p65 and p50 protein levels in various HNSCC cell lines [25]. However, the exact pathological role of NF-κB in chemoresistance still remains controversial [26].

Natural products derived from traditional Chinese medicine (TCM) and Ayurveda practiced in India are gaining increasing importance for the treatment of various cancers in recent years [27, 28]. Garcinol, a poly-isoprenylated benzophenone, is one such compound extracted from the dried rind of the fruit of *Garcinia indica* tree which is used as a traditional folk medicine for the treatment of diseases as diverse as rheumatism, edema, ulcer and infectious diseases [29]. Along with its anti-oxidative, anti-microbial, and anti-inflammatory activities [30–32], anti-neoplastic and chemopreventive roles of garcinol have been identified in variety of cancer cell lines and *in vivo* cancer models, such as leukemia [33], colon cancer [34], breast cancer [35] and oral cancer [36]. Although the mechanisms of garcinol's anti-cancer effects are not fully understood, number of signaling transduction pathways, enzymes and receptors have been implicated to be modulated by garcinol, including FAK [34], NF-κB [35], HAT [37], STAT3 [38, 39] and death receptors [40].

Although garcinol has been previously reported to potentiate TRAIL-induced apoptosis in colorectal cancer [40], there are no prior reports indicating the potential of garcinol as a chemosensitizing agent in HNSCC mouse models. Thus, in the present study, we analyzed whether garcinol could sensitize human HNSCC to cisplatin *in vitro* and in a xenograft mouse model. Our results indicate for the first time that garcinol can indeed inhibit the viability of various HNSCC cell lines, enhance cisplatin-induced apoptosis, and potentiate the anti-tumor activity of cisplatin in a human xenograft HNSCC mouse model through the abrogation of NF-ĸB activation and down-modulation of expression of NF-ĸB-regulated gene products.

## RESULTS

The major goal of this study was to investigate whether garcinol can significantly enhance the anti-cancer effect of chemotherapeutic drug cisplatin in HNSCC and if so, through what molecular mechanism(s).

### Garcinol inhibits the viability and potentiates the apoptotic effect of cisplatin in HNSCC cells *in vitro*

**Figure 1:**
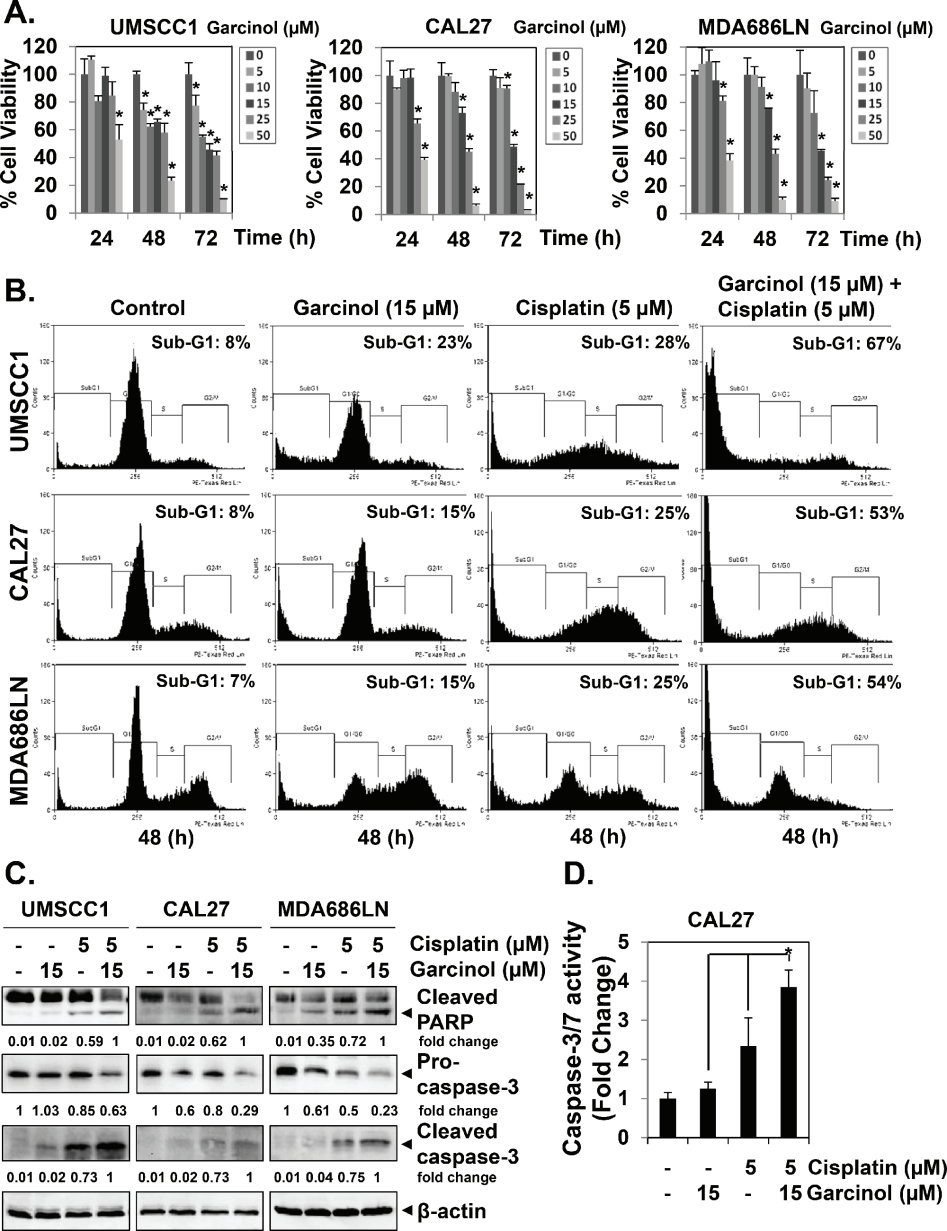
Garcinol suppresses the viability and potentiates the apoptotic effect of cisplatin in HNSCC cells *in vitro* **(A)** Time- and dose-dependent inhibition of cell viability in HNSCC cell lines treated with garcinol. UMSCC1, CAL27, and MDA686LN cells (1 × 10^4^ cells/well) were treated with 0, 5, 10, 15, 25, and 50 μM of garcinol for 24, 48, and 72 h and then subjected to MTT assay. **(B)** Garcinol augments the cisplatin-induced apoptosis in HNSCC. UMSCC1, CAL27, and MDA686LN cells (5 × 10^5^ cells/mL) were treated with 15 μM garcinol and/or 5 μM cisplatin for 48 h, after which the cells were collected and stained with propidium iodide, and subjected to flow cytometric analysis. **(C)** Garcinol enhances the cisplatin-induced activation of caspase-3 and cleavage of PARP. UMSCC1, CAL27, and MDA686LN cells (5 × 10^5^ cells/mL) were treated with 15 μM garcinol and/or 5 μM cisplatin for 48 h, whole-cell extracts were prepared, separated by SDS-PAGE, and subjected to western blot against caspase-3 and PARP antibodies. The same blots were stripped and reprobed with β-actin antibody to verify equal protein loading. **(D)** Garcinol enhances the cisplatin-induced caspase-3/7 activity. CAL27 cells (1 × 10^4^ cells/well) in triplicates were treated with 15 μM garcinol and/or 5 μM cisplatin for 48 h in 96-well plates. Enzymatic activity of caspase-3/7 was determined by Caspase-Glo® 3/7 assay kit. The data is expressed as mean ± SD, compared with the untreated control, (*p < 0.05).

First, the anti-proliferative potential of garcinol was assessed on a panel of diverse HNSCC cell lines. Garcinol inhibited the growth of all three HNSCC cell lines tested (UMSCC1, CAL27 and MDA686LN) in a time- and dose-dependent manner (Figure 1A). The IC50 values at 72 h for HNSCC cell lines are as follows: UMSCC1: 11.6 μM; CAL27: 14.7 μM and MDA686LN: 13.8 μM respectively. Our further analysis of cell viability by MTT assay showed that the combination of garcinol with cisplatin could produce enhanced growth inhibitory effect than either agent used alone (Figure S1A). Interestingly, the combination effects were also found to be synergistic, as assessed by Chou-Talalay method using CompuSyn software (Figure S1B). Next, we investigated whether garcinol can itself induce apoptosis and also potentiate the effect of cisplatin against these cell lines using flow cytometric analysis. Combination of garcinol (15 μM) and cisplatin (5 μM) produced significant apoptosis in all three cell lines, as evidenced by the increase in sub-G1 population, while either agent alone only induced minor apoptotic cell death (Figure 1B). Furthermore, co-treatment of garcinol and cisplatin substantially activated caspase-3 and caused PARP cleavage, as compared to single agent alone (Figure 1C). The enhanced activation of caspase-3 induced by the co-treatment was further verified using quantitative Caspase-Glo® 3/7 assay kit, and the results indicated that the co-treatment of garcinol and cisplatin significantly increased caspase3/7 activity in CAL27 cells (Figure 1D). To evaluate the role of caspase-3 activation in the garcinol-induced apoptosis, the pan-caspase inhibitor Z-VAD-fmk (20 μM) was added to HNSCC cells together with both the drugs for 48 h. As shown in (Figure S2), Z-VAD-fmk treatment completely blocked the increased PARP cleavage, indicating the involvement of caspase-3 in enhancement of apoptosis caused by garcinol. Overall, our results suggest that garcinol can indeed potentiate the apoptotic effects of cisplatin in various HNSCC cells.

### Garcinol suppresses both constitutive and cisplatin-induced NF-κB activation in HNSCC cells

**Figure 2:**
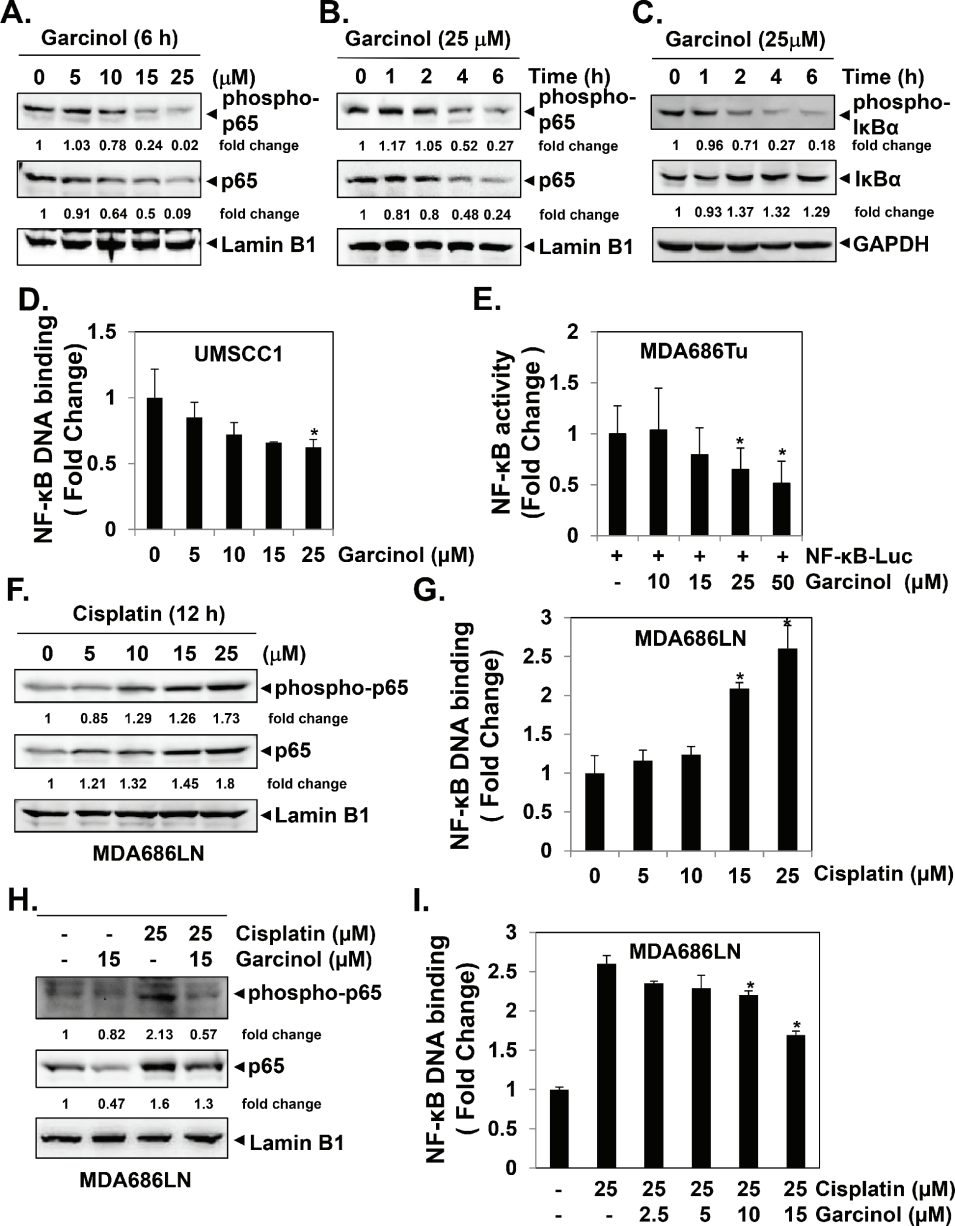
Garcinol suppresses constitutive and inducible NF-κB activation in HNSCC cells **(A–B)** Garcinol inhibits the constitutive p65 phosphorylation and activation in a dose- and time-dependent manner. UMSCC1 cells (1 × 10^6^ cells/mL) were treated with the indicated concentrations of garcinol for 6 h or treated with 25 μM garcinol for the indicated times. Nuclear extracts were prepared and analyzed by western blot using phospho-specific p65 and total p65 antibodies. Membrane was stripped and reprobed for lamin B1 to confirm equal loading. **(C)** Garcinol suppresses phosphorylation and degradation of the constitutive IκBα. UMSCC1 cells (1 × 10^6^ cells/mL) were treated with 25 μM garcinol for 0, 1, 2, 4, and 6 h. Cytoplasmic extracts were prepared and analyzed by western blot using phospho-specific IκBα and total IκBα antibodies. Membrane was stripped and reprobed for glyceraldehyde-3-phosphate dehydrogenase (GAPDH) to confirm equal loading. **(D)** The effect of garcinol on NF-κB DNA-binding activity in HNSCC cells. UMSCC1 cells (1 × 10^6^ cells/mL) were treated with different concentrations of garcinol for 6 h, and nuclear extracts were prepared and assayed for NF-κB activity by ELISA-linked DNA-binding assay. **(E)** Transiently transfected MDA686Tu cells (1 × 10^4^ cells/well) were exposed to different concentrations of garcinol as indicated for 12 h, after which the luciferase activity was determined using Bright-Glo™ luciferase assay kit and was normalized to β-galactosidase activity. (*p < 0.05) **(F–G)** MDA686LN cells (1 × 10^6^ cells/mL) were incubated with different concentrations of cisplatin for 12 h, and nuclear extracts were prepared and subjected to western blot and NF-κB DNA-binding assay. **(H–I)** MDA686LN cells (1 × 10^6^ cells/mL) were treated with different concentrations of cisplatin and garcinol for 12 h, and the nuclear extracts were prepared and subjected to western blot and NF-κB DNA-binding assay.

Next, we explored in detail how garcinol can potentiate the apoptotic effect of cisplatin in HNSCC cell lines. Aberrant NF-κB activation has been shown to contribute to tumor progression and therapeutic resistance in HNSCC [14, 16, 41]. We conducted western blot and NF-κB DNA binding assay to examine whether garcinol can suppress constitutive NF-κB activation in HNSCC cells. We found that garcinol treatment indeed caused the downregulation of constitutive NF-κB expression in a dose- and time-dependent manner (Figure 2A and 2B) and suppression of constitutive phospho-IκBα expression (Figure 2C) as well as NF-κB DNA binding activity (Figure 2D) in HNSCC cells. To next determine whether garcinol affects NF-κB-mediated gene transcription, HNSCC cells were transiently transfected with a plasmid containing NF-κB-regulated luciferase reporter construct, and then incubated with garcinol. Our results indicated that garcinol can significantly abrogate NF-κB-dependent reporter gene expression in a dose-dependent manner in MDA686Tu cells (Figure 2E). We further observed that cisplatin was indeed able to induce NF-κB activation in MDA686LN cells (Figure 2F and 2G), and this cisplatin-induced NF-κB activation could also be significantly abrogated upon garcinol treatment (Figure 2H and 2I). Overall, these data reveal that garcinol can act as a potent suppressor of both constitutive and inducible NF-κB activation in HNSCC cells.

### Garcinol downregulates the expression of various oncogenic proteins in HNSCC cells

**Figure 3:**
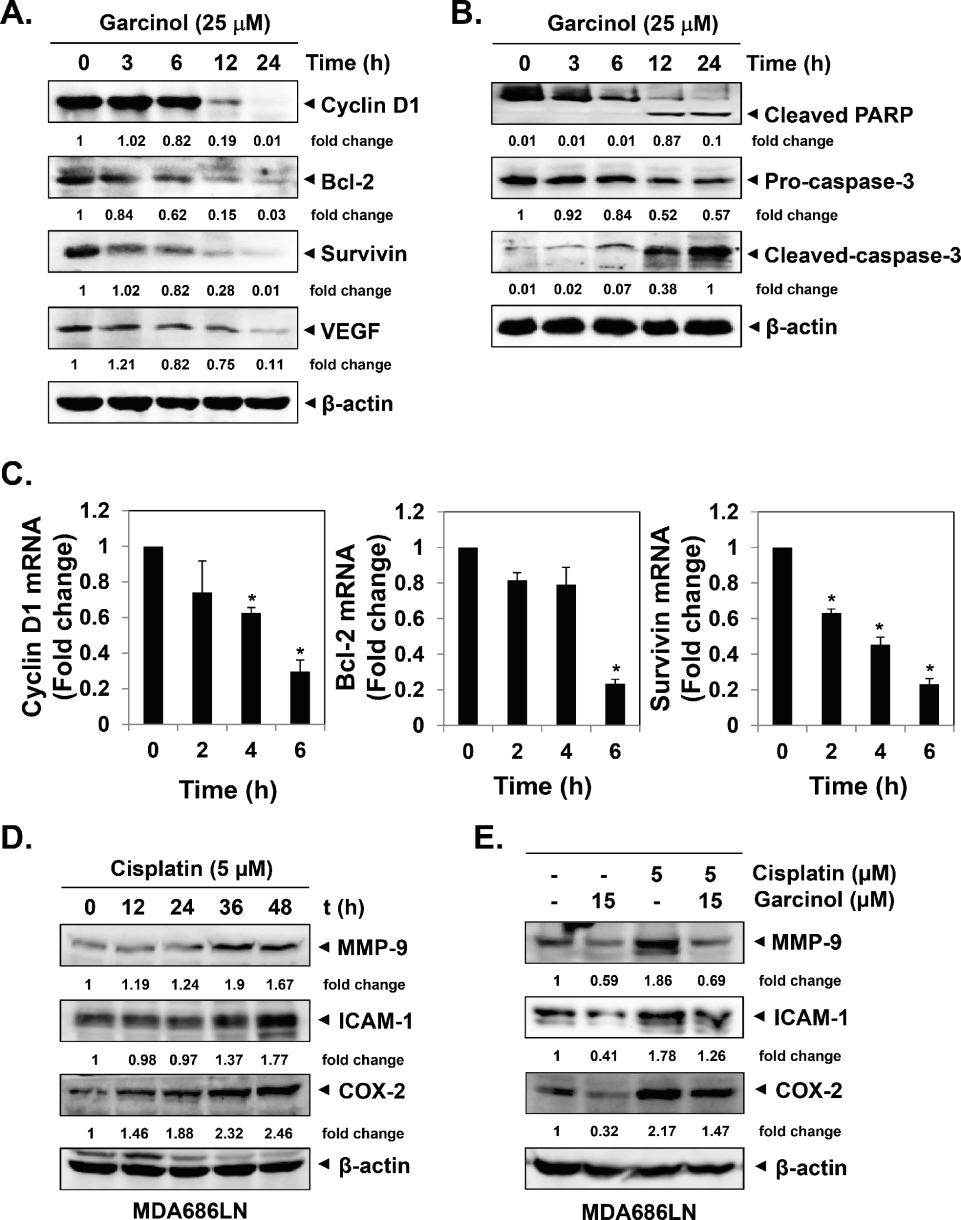
Garcinol suppresses NF-κB-regulated constitutive expression of gene products involved in proliferation, anti-apoptosis and angiogenesis in HNSCC cells **(A–B)** UMSCC1 cells (5 × 10^5^ cells/mL) were treated with 25 μM garcinol for the indicated time points and western blot was performed as described in “Materials and Methods”. **(C)** UMSCC1 cells (5 × 10^5^ cells/mL) were treated with 25 μM garcinol for the indicated time points and real-time PCR analysis was done as described in “Materials and Methods”. (*p < 0.05). **(D)** Cisplatin induces the expression of NF-κB-regulated gene products. MDA686LN cells (5 × 10^5^ cells/mL) were treated with 25 μM garcinol for the indicated time points, and the whole-cell extracts were analyzed by western blot using MMP-9, ICAM-1 and COX-2 antibodies. **(E)** MDA686LN cells (5 × 10^5^ cells/mL) were treated with indicated concentrations of garcinol and/or cisplatin for 48 h, and the whole-cell lysates were prepared and subjected to western blot.

The transcription factor NF-κB regulates the expression of a wide range of genes critical for growth, survival, angiogenesis, metastasis and chemoresistance [42, 43]. Whether garcinol can modulate the expression of these oncogenic gene products was examined by both western blot and real-time PCR analyses. The data showed that garcinol indeed downregulated the expression of proliferative (cyclin D1), anti-apoptotic (Bcl-2, survivin), and angiogenic (VEGF) proteins in a time-dependent manner in UMSCC1 cells (Figure 3A). Furthermore, we found that garcinol also attenuated the expression of *cyclin D1*, *Bcl-2*, and *survivin* genes at transcription level (Figure 3C). And the suppression of NF-κB-regulated anti-apoptotic gene products correlated to the activation of caspase-3 together with the PARP cleavage (Figure 3B). We next also examined the effect of garcinol on the expression of various cisplatin-induced oncogenic proteins in HNSCC cells. We noted that the expression of MMP-9, ICAM-1 and COX-2 increased after cisplatin exposure in a time-dependent manner (Figure 3D), and garcinol treatment was also able to substantially down-modulate cisplatin-induced expression of these oncogenic molecules in HNSCC cells (Figure 3E).

### Garcinol significantly potentiates the anti-tumor effects of cisplatin in HNSCC xenograft model

**Figure 4:**
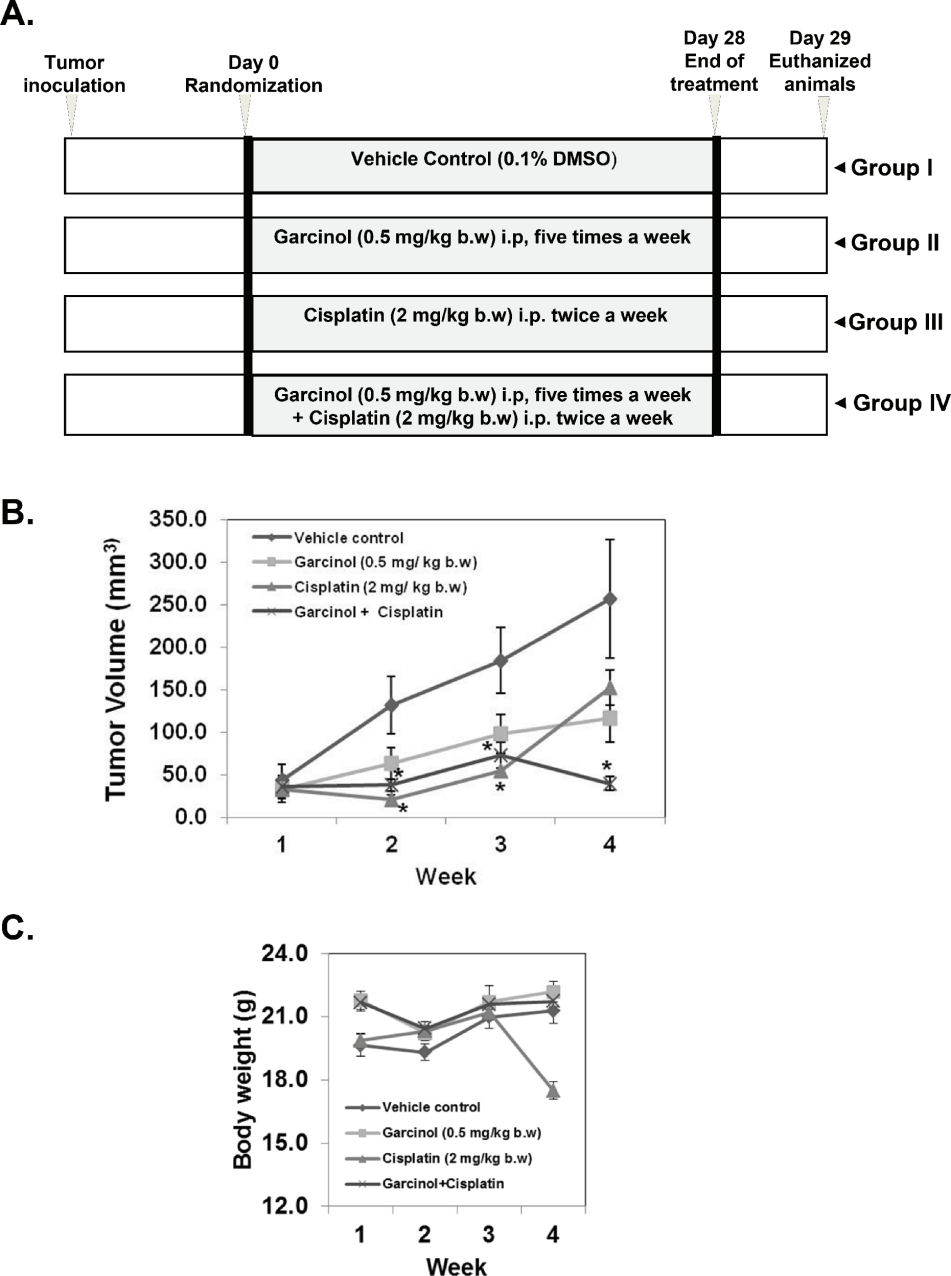
Garcinol potentiates the anti-tumor effect of cisplatin to inhibit the growth of human HNSCC *in vivo* **(A)** A schematic representation of experimental protocol described in “Materials and Methods”. Group I was given 0.1% DMSO (100 μL, i.p.), group II was given garcinol [0.5 mg/kg body weight (b.w.), i.p. five times/week], group III was given cisplatin (2 mg/kg b.w., i.p. twice weekly), and group IV was given garcinol (0.5 mg/kg b.w., i.p. five times/week) and cisplatin (2 mg/kg b.w., i.p. twice weekly). **(B)** Tumor volumes in mice measured during the course of experiment and calculated using the formula V = L × (W)^2^/2. Statistical significance was calculated adapting student's *t*-test, (*p < 0.05). **(C)** Body weight was measured every week during the experimental protocol.

Based on the aforementioned *in vitro* results, we next evaluated the *in vivo* therapeutic potential of garcinol and cisplatin either alone or in combination on the growth of HNSCC CAL27 xenografts in nude mice. A schematic overview of the experimental protocol is presented in (Figure 4A). CAL27 cells were implanted subcutaneously into the right flank of athymic nude mice. When tumors have reached 0.25 cm in diameter after a week, the mice were randomized into 4 groups and started the treatment as per the experimental protocol. The efficacy of the treatment was evaluated by monitoring the tumor volume during the four week treatment. A significant decrease in the tumor volume in single agent treated group was observed from week 2 onwards until the end of the experiment, and the combined treatment exerted more pronounced effect. The tumor volume in the combination of garcinol and cisplatin was significant lower (*p* < 0.05) than garcinol or cisplatin alone group after week 4 (Figure 4B). Body weight of the animals was monitored during the experiment, and no significant loss of body weight was observed in garcinol treated and combination groups (Figure 4C). Together, these data indicate that garcinol can indeed enhance the anti-tumor efficacy of cisplatin *in vivo* without any severe toxicity to the animals.

### Garcinol inhibits CD31 and Ki-67 expression in HNSCC tumor tissues

**Figure 5:**
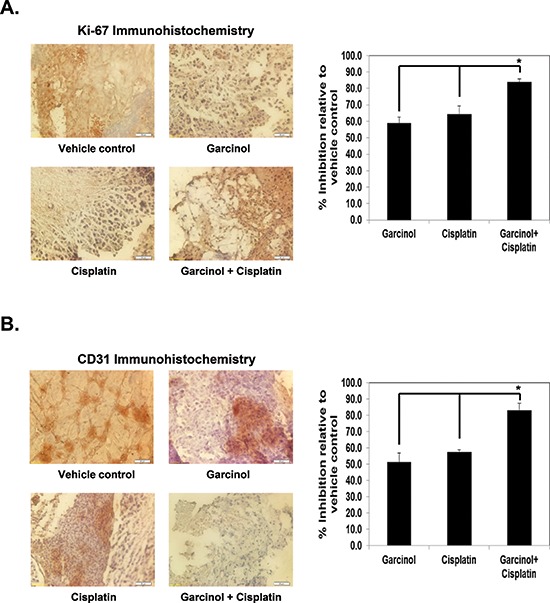
Garcinol enhances the effect of cisplatin against biomarkers of proliferation and angiogenesis in HNSCC cells **(A)** Left panel, immunohistochemical analysis of proliferation marker Ki-67 indicates the inhibition of HNSCC cell proliferation following garcinol treatment either alone or in combination with cisplatin-treated groups of animals. A, Right panel, quantification of Ki-67 + cells as described in “Materials and Methods”. Columns, mean of triplicate; bars, SE, (*p < 0.01). **(B)** Left panel, immunohistochemical analysis of CD31 for microvessel density in HNSCC tumors indicates the inhibition of angiogenesis by either garcinol alone and in combination with cisplatin. **(B)** Right panel, quantification of CD31 + microvessel density as described in “Materials and Methods”. Columns, mean of triplicate; bars, SE, (*p < 0.01).

Since Ki-67 is used as a cellular marker for proliferation and CD31 index is considered as a biomarker for microvessel density, we carried out immunohistochemical analysis to evaluate whether garcinol and cisplatin treatment can modulate the expression of these biomarkers to manifest their effects against HNSCC. (Figure 5A) showed that both garcinol and cisplatin downregulated the expression of Ki-67 in tumor tissue to the similar extent, and the two together were more effective (*p* < 0.01 versus garcinol alone; *p* < 0.01 versus cisplatin alone). Similarly, both agents significantly inhibited CD31 expression alone, and the maximum decrease was noted when the two drugs were used in combination (*p* < 0.01 versus garcinol alone; *p* < 0.01 versus cisplatin alone; (Figure 5B).

### Garcinol attenuates the constitutively activated NF-κB and its-regulated gene products in HNSCC tumor tissues

**Figure 6:**
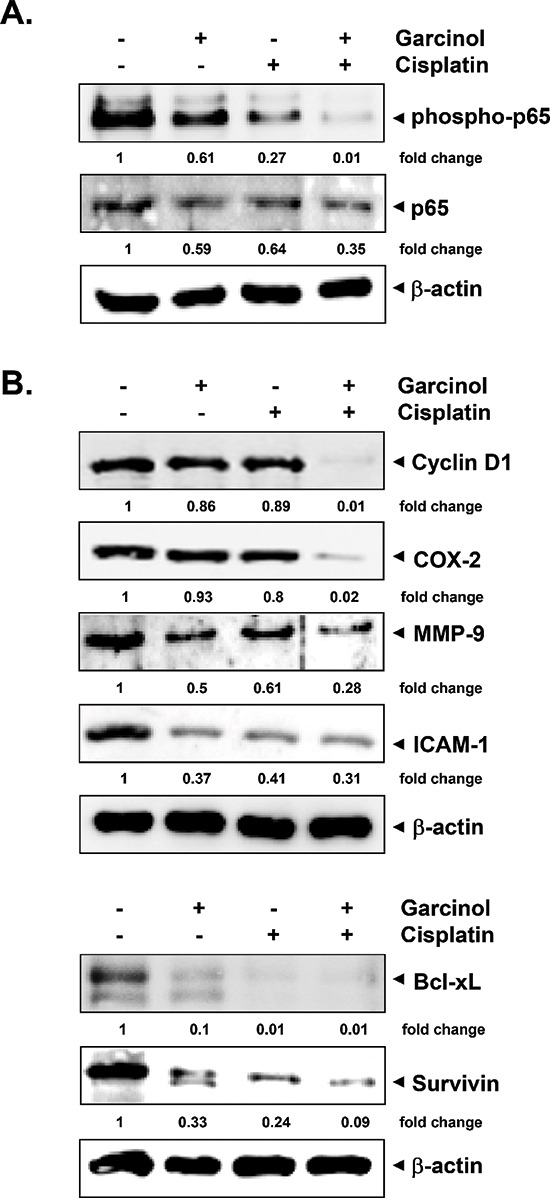
Garcinol suppresses the activation of NF-κB and expression of NF-κB-regulated gene products in HNSCC tumor tissue samples **(A)** Western blot analysis shows the inhibition of constitutive phospho-p65 and NF-κB (p65) by garcinol in the nuclear extracts from tumor tissues. **(B)** Western blotting analysis shows that combination of garcinol and cisplatin inhibited the expression of NF-κB-dependent gene products cyclin D1, COX-2, MMP-9, ICAM-1, Bcl-xL and survivin in HNSCC tumor tissues. Samples from 3 mice in each group were analyzed and representative data are shown.

**Figure 7:**
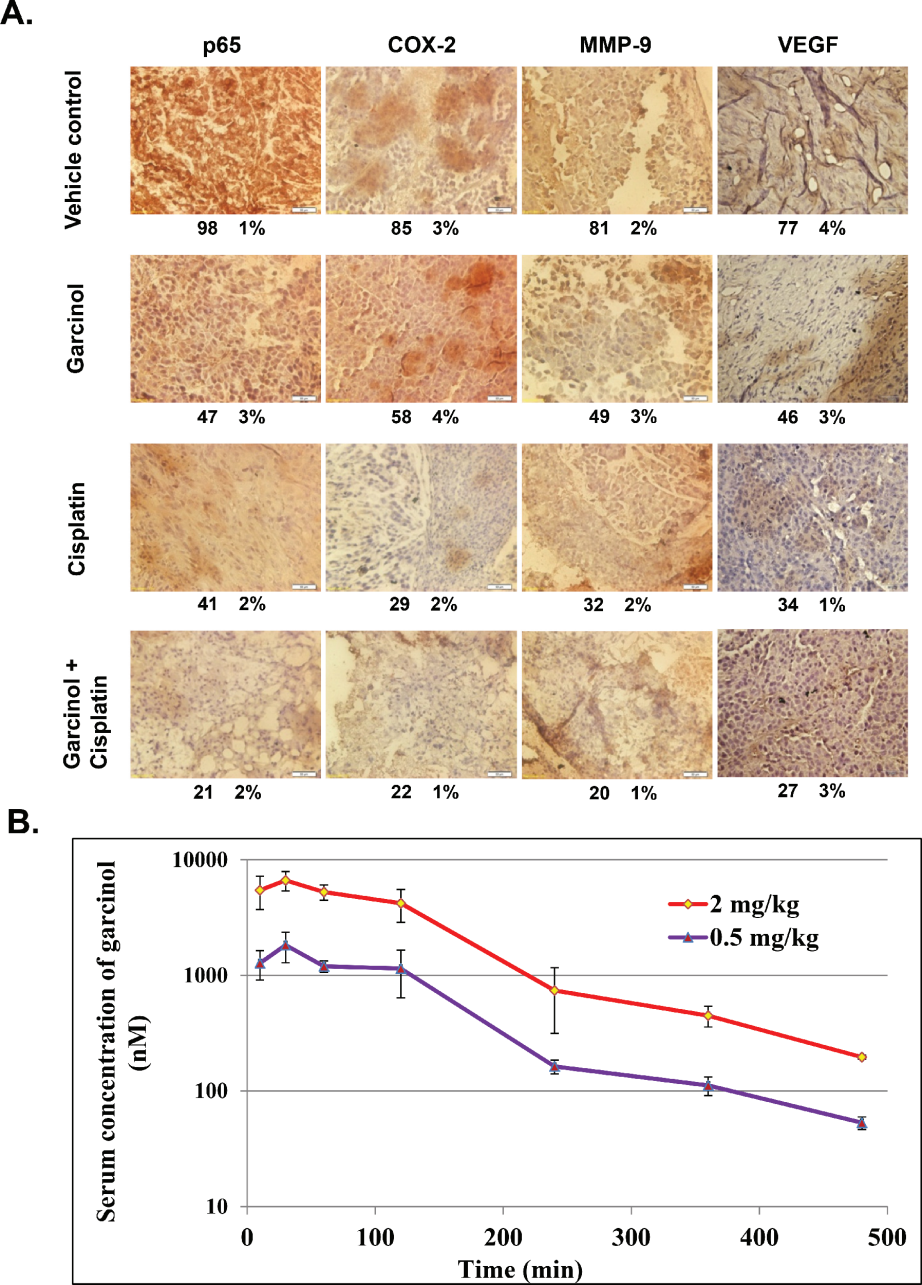
**(A)** Immunohistochemical analysis of p65, COX-2, MMP-9 and VEGF shows significant inhibition of these biomarkers by either garcinol alone or in combination with cisplatin in HNSCC tumor tissues with percentage positive staining for the given biomarker. **(B)** Athymic nu/nu female mice were dosed with 0.5 mg/kg and 2 mg/kg of garcinol, the serum concentrations of the drug were assessed at 10 min, 30 min, 1 h, 2 h, 4, 6, and 8 h post dose. The error bars represent the mean ± SD.

We next investigated whether the observed chemosensitizing effects of garcinol in mice were mediated through the inhibition of NF-κB activation. Western blot analysis of nuclear extracts obtained from tumor tissues showed that garcinol either alone or in combination with cisplatin effectively suppressed the constitutive phospho-p65 and NF-κB (p65) expression in HNSCC tumor tissues (Figure 6A). NF-κB is known to regulate the expression of proliferative (cyclin D1, COX-2), invasive/metastatic (MMP-9, ICAM-1) and anti-apoptotic (Bcl-xL, survivin) proteins [44, 45]. Accordingly, we performed western blot analysis to examine whether garcinol and cisplatin can modulate the expression of these NF-κB-regulated gene products in HNSCC tumor tissues. (Figure 6B) clearly showed that combination treatment of garcinol and cisplatin was very effective in downregulating the expression of various oncogenic gene products involved in HNSCC growth, survival, invasion, and metastasis. Furthermore, the results of immunohistochemical analysis on human HNSCC tumor samples demonstrated that garcinol in combination with cisplatin significantly suppressed NF-κB (p65), COX-2, VEGF, and MMP-9, and such downregulation was more impressive than either garcinol or cisplatin alone (Figure 7A). The immunohistochemical analysis results further support the observation of western blot analysis. Taken together, our data indicate that garcinol suppresses constitutive NF-κB activation thereby attenuating the expression of genes involved in proliferation, survival, invasion, and angiogenesis *in vivo*.

### Pharmacokinetic profile of garcinol

The pharmacokinetic profile of garcinol at doses of 0.5 and 2 mg/kg are presented in (Figure 7B). Each point represents a mean value of three serum concentrations. After i.p. administration, garcinol was quickly absorbed into the bloodstream and reached a peak serum concentration (Cmax) of 1825.4 and 6635.7 nM at 0.5 h post-dose for dose of 0.5 and 2 mg/kg, respectively. In addition, the area under the serum concentration-time curve (AUC) at 8 h is increased proportionally from 4298.3 to 16944.8 h*nM.

## DISCUSSION

Despite the recent advances in surgery and radiotherapy, and development of sophisticated systemic therapy, limited improvement in treating metastatic HNSCC has been achieved. Two-thirds of all patients present advanced stage III or IV tumors with low locoregional control rates and 5-year survival rates below 50% [46, 47]. Thus, novel agents that are non-toxic, efficacious, and can significantly enhance the effects of existing chemotherapeutic drugs are urgently needed. The present study was designed to explore the potential of garcinol, a polyisoprenylated benzophenone, as a chemosensitizing agent in combating HNSCC. We found that garcinol inhibited the viability of various HNSCC cells, enhanced the apoptotic effect of cisplatin in a panel of head and neck cancer cell lines, negatively regulated both constitutive and inducible NF-κB activation and further suppressed the expression of NF-κB-regulated gene products. Furthermore, we observed that garcinol can effectively suppress the tumor growth alone and in combination with cisplatin in a xenograft mouse model by down-modulating various proliferative and inflammatory biomarkers.

The anti-proliferative effects of garcinol have been previously reported in several cancers, for example, treatment of garcinol caused significant reduction in cell viability in leukemia [33] and pancreatic adenocarcinoma cells [48], and the inhibitory effects were correlated with the DNA fragmentation and cell cycle disruption. However, whether garcinol exhibits similar activity in HNSCC cell lines has never been evaluated before. In our study, we first demonstrated that garcinol inhibited the viability of HNSCC cell lines in a time- and dose-dependent manner. We also found that garcinol suppressed the expression of an important cell cycle regulator cyclin D1, which may explain its observed anti-proliferative effect in HNSCC cells. We further report for the first time that garcinol when used in combination with cisplatin, was highly effective in inducing substantial apoptosis in HNSCC cell lines. This is quite intriguing because although our group has already recently reported that garcinol can alone induce apoptosis in HNSCC cells [39], potential use of garcinol in combination with existing chemotherapeutic agents like cisplatin has never been investigated in HNSCC cells. We hypothesize that this effect may be attributed to the downregulation of multiple cell survival proteins such as Bcl-2 and survivin. Interestingly, our results are in a part agreement with recent studies, in which two curcumin analogues (H-4073 and FLLL32) [49, 50] were reported to enhance the therapeutic efficacy of cisplatin treatment in HNSCC through the abrogation of STAT3 signaling axis that has also been reported by us recently to be negatively regulated by garcinol [39].

Growing evidences have indicated that acquired resistance to cisplatin in tumor cells is associated with multiple molecular mechanisms [51–55], among which is the activation of NF-κB. Prior studies reported that cisplatin could induce NF-κB activation in different type of cancers, which led to the drug resistance [21, 56, 57]. Interestingly, we found that cisplatin can substantially increase nuclear p65 expression and phosphorylation level as well as NF-κB DNA binding activity in HNSCC cells, and both the constitutive and cisplatin-induced NF-κB activation could be suppressed upon garcinol treatment in HNSCC cells. A recent report has suggested that NF-κB can also mediate cisplatin-induced resistance through histone modifications in HNSCC cells [57], and thus inducible NF-κB inhibitory effects of garcinol may be mediated through its previously documented potent histone acetyltransferases blocking effects as observed in other tumor cells [37]. Our results are also in part agreement with another study in which curcumin was also found to enhance the effect of cisplatin in suppression of HNSCC via inhibition of IKKβ protein of the NF-κB pathway [58]. Tumor cells are known to secrete substantial amounts of matrix metalloproteinases, which not only degrade extracellular matrix (ECM) but also facilitate tumor cell migration and invasion to neighboring tissues [59]. The cellular adhesion molecule, ICAM-1 has also been implicated in various stages of tumor progression and metastasis [60], whereas COX-2 contributes to different stages of cancer development including uncontrolled growth, metastasis and angiogenesis [61, 62]. We further observed that cisplatin also substantially induced the expression of the MMP-9, ICAM-1 and COX-2 proteins, and garcinol treatment was able to suppress both constitutive and cisplatin-induced expression of above mentioned NF-κB-regulated gene products involved in HNSCC progression.

In order to examine the efficacy of garcinol in a xenograft mouse model, a pharmacokinetic study of garcinol was conducted to define suitable doses for treatment of mice in pharmacodynamics study. Two doses of garcinol (0.5 and 2 mg/kg) were used for evaluation of pharmacokinetic property of garcinol through intraperitoneal administration. The pharmacokinetic results indicated that garcinol showed a sufficient systemic exposure and good dose proportionality which is an important property for garcinol in its translational development from animal to human subjects. The pharmacokinetic results suggested that 0.5 and 2 mg/kg of garcinol are the suitable doses for its pharmacodynamics evaluation. These hypotheses had been confirmed by our xenograft mouse experiment in which garcinol at 0.5 mg/kg dose was found to inhibit the tumor growth effectively. We observed that garcinol or cisplatin alone reduced tumor volume by 50%, and the combination of the two agents augmented the effect to a significant greater extent. We also noticed that the garcinol suppressed the expression of both proliferative and microvessel density biomarkers (Ki-67 and CD31) respectively. Further analyses on tumor tissues by western and IHC showed that garcinol downregulated the expression of critical oncogenic molecules involved in proliferation (cyclin D1, COX-2), survival (Bcl-xL, survivin), angiogenesis (VEGF) and invasion (MMP-9, ICAM-1) and all of these effects were further enhanced upon cisplatin treatment.

Garcinol has been previously tested in pancreatic cancer and breast cancer, in conjunction with other anti-cancer agents namely gemcitabine, curcumin and anti-ERBB2 antibody [63–65]. There are also prior animal studies on garcinol as chemopreventive agent against nitroquinoline 1-oxide (4-NQO)-induced tongue cancer in rats [36] and 7, 12-dimethylbenz [*a*] anthracene (DMBA)-induced oral cancer in hamster cheek pouch [66]. However, the effects of garcinol in combination with anti-cancer therapies in HNSCC mouse models have never been studied before. Our results indicate for the first time that garcinol indeed exerts its chemosensitizing effects in HNSCC by down-modulating the activation of NF-κB and various proliferative and inflammatory biomarkers both *in vitro* and *in vivo*. Overall, based on our novel findings, well designed clinical trials are needed to analyze the therapeutic efficacy of garcinol either alone or in combination with chemotherapy in HNSCC patients.

## MATERIALS AND METHODS

### Reagents

Cisplatin, MTT, Tris, glycine, NaCl, sodium dodecyl sulfate (SDS), Tween-20, bovine serum albumin (BSA), and β-actin antibody were purchased from Sigma-Aldrich. Garcinol with purity more than 98% was prepared from *Garcinia indica* fruit rind as described previously [37]. Garcinol was dissolved in dimethyl sulfoxide (DMSO) as a 50 mM stock solution and stored at −20°C, and cisplatin was dissolved in distilled water as a 3.33 mM stock solution and stored at room temperature for the experiments. Further dilutions were done in cell culture medium. Dulbecco's modified Eagle medium (DMEM), fetal bovine serum (FBS), non-essential amino acid (NEAA), sodium pyruvate, vitamin and L-glutamine, trypan blue vital stain, and antibiotic-antimycotic mixture were obtained from Life Technologies. Antibodies against p65, IκBα, cyclin D1, Bcl-2, survivin, VEGF, caspase-3, PARP, Bcl-xL, Ki-67, ICAM-1, COX-2, goat anti-rabbit-horseradish peroxidase (HRP) conjugate, goat anti-mouse HRP and Z-VAD-fmk were obtained from Santa Cruz Biotechnology. Antibodies to phospho-specific p65 (Ser 536), phospho-specific IκBα (Ser 32), lamin B1, MMP-9 and CD31 were purchased from Cell Signaling Technology. Bradford reagent was purchased from Bio-Rad. Nuclear extraction kit and DNA-binding kit was obtained from Active Motif. Caspase-Glo® 3/7 and Bright-Glo™ luciferase assay kits were purchased from Promega. Lipofectamine® 2000 and TRIzol® reagent were purchased from Life Technologies.

### Cell lines

Human HNSCC cell line UMSCC1 was kindly provided to us by Prof. Thomas E. Carey (University of Michigan, Ann Arbor, MI) and has been characterized previously. MDA686LN and MDA686Tu cells were kindly provided by Prof. Jeffrey N. Myers (The University of Texas MD Anderson Cancer Center, Houston, Texas) and has been characterized previously. CAL27 was purchased in the year 2009 from American Type Culture Collection. UMSCC1 and CAL27 were cultured in DMEM supplemented with 10% FBS, 100 U/mL penicillin, and 100 μg/mL streptomycin, MDA686LN and MDA686Tu cells were cultured in DMEM supplemented with 10% FBS, 100 U/mL penicillin, 100 μg/mL streptomycin,1 × NEAA, 1 × sodium pyruvate, 1 × vitamin and 2 mM glutamine.

### Western blotting

Vehicle or drug-treated whole-cell extracts were lysed in lysis buffer [250 mM NaCl, 20 mM HEPES, 2 mM EDTA (pH 8.0), 0.5 mM EGTA, 0.1% Triton X-100, 1.5 μg/mL aprotinin, 1.5 μg/mL leupeptin, 1 mM phenylmethylsulfonylfluoride (PMSF), and 1.5 mM NaVO_4_]. Lysates were then spun at 13,300 rpm for 10 min to remove insoluble material and resolved on a 10% SDS gel. After electrophoresis, the proteins were electrotransferred to a nitrocellulose membrane (Bio-Rad), blocked with Blocking One (Nacalai Tesque, Inc.), and probed with antibodies of interest overnight at 4ºC. The blot was washed with TBS with 0.1% Tween-20, exposed to HRP-conjugated secondary antibodies for 1 h, and finally examined by chemiluminescence using Western Bright Sirius HRP substrate (Advansta).

### Cell viability assay

The inhibitory effect of garcinol against HNSCC cell growth was determined by the MTT dye uptake method as described previously [39]. Briefly, the cells (5 × 10^4^ cells/mL) were incubated in triplicate in a 96-well plate in the presence or absence of different concentrations of garcinol in a final volume of 0.2 mL for indicated time intervals at 37ºC. Thereafter, 20 μL MTT solution (5 mg/mL in PBS) was added to each well. After a 2 h incubation at 37°C, 0.1 mL lysis buffer (20% SDS, 50% dimethylformamide) was added after removal of the medium, incubation was continued at 37°C for 1 h, and then the optical density (OD) at 570 nm was measured by the Safire2™ microplate reader (Tecan).

### Flow cytometric analysis

To determine the effect of garcinol on the cell cycle, HNSCC cells (5 × 10^5^ cells/mL) were seeded in a 6-well plate and then exposed to single agent or combination of garcinol and cisplatin for 48 h. Thereafter, cells were trypsinized, washed with PBS, and fixed with 70% ethanol for 30 min on ice. Cells were then washed again, resuspended, and stained in PBS containing 10 μg/mL propidium iodide (PI) and 1 μg/mL RNase A for 30 min at room temperature. Cell distribution across the cell cycle was analyzed with a CyAn ADP flow cytometer (Dako Cytomation).

### Caspase-Glo® 3/7 luminescent assay

Caspase activity was measured using Caspase-Glo® 3/7 assay kit (Promega) according to the manufacturer's instructions. HNSCC cells (1 × 10^4^ cells/well) in 96-well plate were treated with single agent or combination of garcinol and cisplatin as indicated for 48 h. Equal volume of Caspase-Glo® 3/7 Reagent was then added to the wells to provide the 1:1 ratio of reagent volume to sample volume. After incubation for 1 h at room temperature, the luminescence was measured by Tecan microplate reader.

### NF-κB luciferase reporter assay

MDA686Tu cells (1 × 10^4^ cells/well) in 96-well were transfected of plasmid with NF-κB responsive plate elements linked to a luciferase reporter gene using lipofectamine® 2000. After transfection for 48 h, indicated concentrations of garcinol were added into the wells for 12 h. Luciferase activity was determined using Bright-Glo™ luciferase assay kit from Promega.

### RNA extraction and real time PCR analysis

Total RNA was extracted using the TRIzol reagent (Life Technologies), according to the manufacturer's instructions. Reverse transcription was then carried out as described previously [67]. For a 50 μL reaction, 10 μL of RT product was mixed with 1 × Taq-Man^®^ Universal PCR Master mix, 2.5 μL of 20 × TaqMan probes for cyclin D1, Bcl-2, and survivin, respectively, 2.5 μL of 20 × 18S RNA TaqMan probe as the endogenous control for each targeting gene and topped up to 50 μL with sterile water. A negative control for RT, in which sterile water replaced the RNA template, was included. Another control, where RT mix was replaced with sterile water, was included to check for DNA contamination. Real time PCR was done using the 7500 Fast Real-Time PCR System (ABI PRISM 7500; Applied Biosystems, Foster City, CA) with the following protocol: 50°C for 2 min, 95°C for 10 min, followed by 40 cycles of denaturing at 95°C for 15 s and extension at 60°C for 1 min. The results were analyzed using Sequence Detection Software version 1.3 provided by Applied Biosystems. Relative gene expression was obtained after normalization with endogenous human 18 S and determination of the difference in threshold cycle (Ct) between treated and untreated cells using the 2-ΔΔ Ct method. Primers and probes for human cyclin D1, Bcl-2, and survivin were purchased as kits from Applied Biosystems (Assays-on-Demand).

### HNSCC xenograft mouse model

All procedures involving animals were reviewed and approved by NUS Institutional Animal Care and Use Committee. Six-week old athymic nu/nu female mice (Animal Resource Centre, Australia) were implanted subcutaneously in the right flank with CAL27 cells (3 × 10^6^ cells/100 μL saline). When tumors have reached 0.25 cm in diameter, the mice were randomized into the following treatment groups (n = 5/group): (I) untreated control (0.1% DMSO, 100 μL daily); (II) garcinol (0.5 mg/kg body weight, suspended in 0.1% DMSO, intraperitoneal [i.p.] injection, five times/week); (III) cisplatin alone (2 mg/kg body weight, suspended in 0.1% DMSO, i.p. injection, twice/week); and (IV) combination (garcinol, 0.5 mg/kg body weight, i.p. injection, five times/week and cisplatin, 2 mg/kg body weight, i.p. injection, twice/week). Therapy was continued for 4 weeks, and the animals were euthanized 24 h after the last dose. Primary tumors were excised and the final tumor volume was calculated using the formula V = L × (W)^2^/2 where L is the length and W is the width. Half of the tumor tissue was fixed in formalin and embedded in paraffin for immunohistochemistry (IHC) analysis. The other half was snap frozen in liquid nitrogen and stored at −80°C.

### Immunohistochemical analysis of tumor samples

Solid tumors from control and drug-treated groups were fixed with 10% phosphate buffered formalin, processed, and embedded in paraffin. The sections were cut and deparafinized in xylene, dehydrated in graded alcohol, and finally hydrated in water. Antigen retrieval was conducted by boiling the slide in 10 mM sodium citrate (pH 6.0) for 30 min. Immunohistochemistry was conducted following the manufacturer's instructions (Dako LSAB Kit). Briefly, endogenous peroxidases were quenched with 3% hydrogen peroxide. Nonspecific binding was blocked by incubation in the blocking reagent in the LSAB Kit. Sections were incubated overnight with primary antibodies as follows: anti-Ki-67, anti-CD31, anti-COX-2, anti-MMP-9, anti-p65, and anti-VEGF (each at 1:100 dilutions). The slides were subsequently washed in TBS-T and were incubated with biotinylated linker for 30 min, followed by incubation with streptavidin conjugate. Immunoreactive species were detected using 3,30′-diaminobenzidine tetrahydrochloride as a substrate. The sections were counterstained with Gill's hematoxylin and mounted under glass cover slips. Images were taken using an Olympus BX51 microscope (magnification, × 40). Quantitative analysis of immunohistochemistry images were done by visual scores between the control and treated images. In this expression quantitation technique, each image is divided into four parts and each part is individually quantitated for the biomarker expression. A cell scored as positive refers simply to the presence of brown staining (peroxidase) in any part of the studied tissue. A negative cell scored refers to no staining or weak staining.

### Preparation of cytoplasmic and nuclear extract

Nuclear proteins were extracted from cell lines using TransAM Nuclear Extract Kit according to the manufacturer's instructions. Briefly, cells were washed with phosphatase inhibitors-containing PBS and collected by scraping. After centrifugation at 500 rpm for 5 min, the supernatant was discarded and the cell pellet was resuspended with 1 × hypotonic buffer, after which was incubated on ice for 15 min. Detergent was added to the suspension and vortexed at highest setting for 10 seconds. Supernatant (cytoplasmic fraction) was collected after centrifugation and stored at −80ºC. The nuclear pellet was resuspended in complete lysis buffer by pipetting up and down several times. After vortexing for 10 seconds, the suspension was incubated for 30 min on ice on a rocking platform set at 150 rpm. Suspension was then vortexed again for 30 seconds and centrifuged at 14,000 g for 10 min. Supernatant (nuclear fraction) was collected and stored at −80°C until ready to use.

### Measurement of NF-κB activation in HNSCC cell lines and tumor samples

To determine NF-κB activation, we conducted DNA-binding assay using TransAM NF-κB Kit according to the manufacturer's instructions and as previously described [39]. Briefly, 20 μg of nuclear proteins were added into a 96-well plate coated with an unlabeled oligonucleotide containing the consensus binding site for NF-κB (5′-GGGACTTTCC-3′) and incubated for 1 h. The wells were washed and incubated with antibodies against NF-κB p65 subunit for 1 h. An HRP-conjugated secondary antibody was then applied to detect the bound primary antibody and it provided the basis for colorimetric quantification. The enzymatic product was measured at 450 nm with a reference wavelength of 655 nm by microplate reader.

### Pharmacokinetics study of garcinol in mice

Pharmacokinetics of garcinol was conducted in nude mice by i.p. injection at dose of 0.5 mg/kg or 2 mg/kg. The mice were allowed food and water ad libitum before pharmacokinetics experiment. About 150 μL of blood was taken from facial vein of mice at 10 min, 30 min, 1, 2, 4, 6, and 8 h post-dose. After centrifugation of blood samples at 14,000 rpm at 4°C for 10 min, mouse sera (the supernatant) were transferred into Eppendorf tube. 10 μL of each serum was processed by adding 3-folds excess of acetonitrile-containing isogarcinol, the internal standard followed by vortexing for 1 min. The tube was then centrifuged at 14,000 rpm at 4°C for 10 min. Then 30 μL of the supernatants were transferred into glass inserts (250 μL) with 70 μL of 10 mM ammonia acetate solution. 50 μL of processed serum samples were injected into LC-MS/MS which was carried out under negative electrospray ionization (ESI) and multiple reaction monitoring (MRM) mode. Mass spectra of garcinol and its internal standard, isogarcinol showed a same precursor ion [M–H]^−^ at m/z of 601. The daughter ion monitored for garcinol and isogarcinol was 409 and 335, respectively. SCIEX Analyst software (version 1.4.2) was used for data acquisition and analysis.

### Statistical analysis

Statistical analysis was performed by Student's *t*-test and one way analysis of variance, (ANOVA). A *p* value of less than 0.05 was considered statistically significant. AbbreviationsHNSCC, head and neck squamous cell carcinoma; NF-κB, nuclear factor kappa B; COX-2, cyclooxygenase-2; MMP-9, matrix metalloproteinase 9 ICAM-1, intercellular adhesion molecule 1, FAK, focal adhesion kinase HAT, histone acetyltransferase, STAT3, signal transducer and activator of transcription 3, IκBα, inhibitor of NF-κB alpha, VEGF, vascular endothelial growth factor, IKKβ, IκB kinase beta

## SUPPLEMENTARY FIGURES



## Abbreviations

HNSCC: head and neck squamous cell carcinoma
NF-κB: nuclear factor kappa B
COX-2: cyclooxygenase-2
MMP-9: matrix metalloproteinase 9
ICAM-1: intercellular adhesion molecule 1
FAK: focal adhesion kinase
HAT: histone acetyltransferase
STAT3: signal transducer and activator of transcription 3
IκBα: inhibitor of NF-κB alpha
VEGF: vascular endothelial growth factor
IKKβ: IκB kinase beta
