# Supplementary material for: Garcinol sensitizes human head and neck carcinoma to cisplatin in a xenograft mouse model despite downregulation of proliferative biomarkers
Source: Oncotarget. 2015 Feb 28;6(7):5147–63. doi: 10.18632/oncotarget.2881 (PMC4467139; doi:10.18632/oncotarget.2881)
Supplement: Supplementary file 1 [file oncotarget-06-5147-s001.pdf]

SUPPLEMENTARY FIGURES

A.

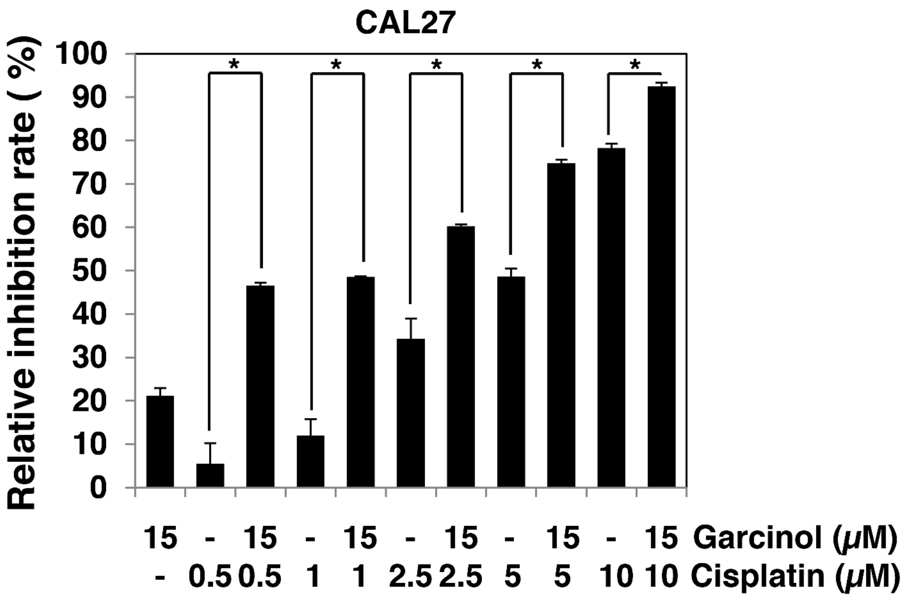

B.

Dose of garcinol (μM)	Dose of Cisplatin (μM)	CI Value
15.0	0.5	0.15995
15.0	1.0	0.26651
15.0	2.5	0.43118
15.0	5.0	0.51447
15.0	10.0	0.35462

Supplementary Figure 1: **(A)** Garcinol augments the cytotoxic effect of cisplatin in HNSCC cells. CAL27 cells (1×10^4 cells/well) were exposed for 48 h to different concentrations of garcinol and cisplatin, and their combination and then subjected to MTT assay to analyze the viability of tumor cells. **(B)** Assessment on the type of combination relationship was done using the method developed by Chou-Talalay multiple drug effect equation. The degree of synergy was assessed based on CI values, where CI values of > 1.0 implies antagonism, 1.0 implies additive and < 1.0 implies synergistic effect relationships.

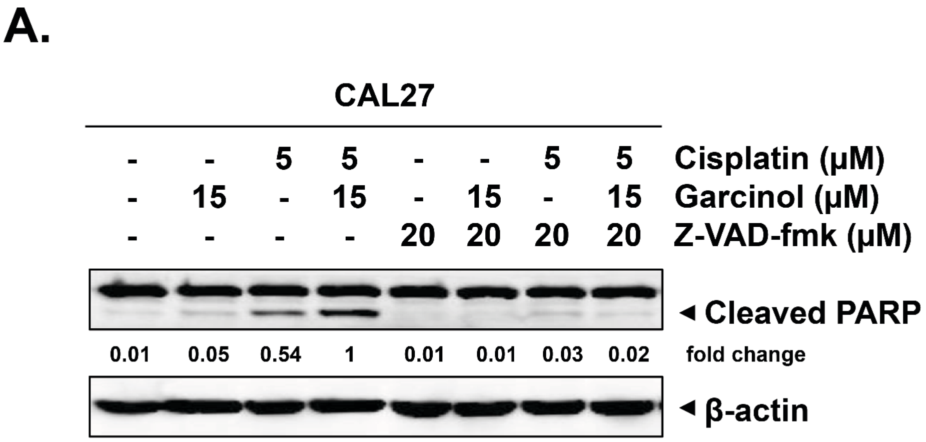

Supplementary Figure 2: Z-VAD-fmk abrogates the garcinol-induced caspase-3 activation. CAL27 cells (5×10^5 cells/mL) were treated with 15 μM garcinol and/or 5 μM cisplatin for 48 h in the absence or presence of 20 μM Z-VAD-fmk, whole-cell extracts were prepared, separated by SDS-PAGE, and subjected to western blot against PARP antibody. The same blot was stripped and reprobed with β-actin antibody to verify equal protein loading.
